# Comparison of Two Highly Discriminatory Typing Methods to Analyze *Aspergillus fumigatus* Azole Resistance

**DOI:** 10.3389/fmicb.2018.01626

**Published:** 2018-07-20

**Authors:** Rocio Garcia-Rubio, Pilar Escribano, Ana Gomez, Jesus Guinea, Emilia Mellado

**Affiliations:** ^1^Mycology Reference Laboratory, National Centre for Microbiology, Instituto de Salud Carlos III, Madrid, Spain; ^2^Department of Clinical Microbiology and Infectious Diseases, Hospital General Universitario Gregorio Marañón, Madrid, Spain; ^3^Instituto de Investigación Sanitaria Gregorio Marañón, Madrid, Spain; ^4^Department of Medicine, School of Medicine, Complutense University of Madrid, Madrid, Spain

**Keywords:** *Aspergillus fumigatus*, molecular typing, genotypic analysis, STR*Af*, TRESPERG, azole resistance

## Abstract

*Aspergillus fumigatus* molecular typing has become increasingly more important for detecting outbreaks as well as for local and global epidemiological investigations and surveillance. Over the years, many different molecular methods have been described for genotyping this species. Some outstanding approaches are based on microsatellite markers (STR*Af* assay, which is the current gold standard), or based on sequencing data (TRESP typing improved in this work with a new marker and was renamed TRESPERG). Both methodologies were used to type a collection of 212 *A. fumigatus* isolates that included 70 azole resistant strains with diverse resistance mechanisms from different geographic locations. Our results showed that both methods are totally reliable for epidemiological investigations showing similar stratification of the *A. fumigatus* population. STR*Af* assay offered higher discriminatory power (*D* = 0.9993) than the TRESPERG typing method (*D* = 0.9972), but the latter does not require specific equipment or skilled personnel, allowing for a prompt integration into any clinical microbiology laboratory. Among azole resistant isolates, two groups were differentiated considering their resistance mechanisms: *cyp51A* single point mutations (G54, M220, or G448), and promoter tandem repeat integrations with or without *cyp51A* modifications (TR_34_/L98H, TR_46_/Y121F/A289T, or TR_53_). The genotypic differences were assessed to explore the population structure as well as the genetic relationship between strains and their azole resistance profile. Genetic cluster analyses suggested that our *A. fumigatus* population was formed by 6–7 clusters, depending on the methodology. Also, the azole susceptible and resistance population showed different structure and organization. The combination of both methodologies resolved the population structure in a similar way to what has been described in whole-genome sequencing works.

## Introduction

*Aspergillus fumigatus* is a worldwide saprotrophic mold that produces very large numbers of airborne spores (Latgé, 1999). It is the primary and most predominant *Aspergillus* human-pathogenic species and therefore the most common cause of aspergillosis (Kontoyiannis et al., 2010; Pappas et al., 2010). Triazoles are the first line drugs used for treating aspergillosis. However, the emergence of azole resistance in *A. fumigatus* is increasing worldwide, limiting the clinical efficacy of azoles in both clinical and environmental settings (Verweij et al., 2016). Up to now, most azole resistance mechanisms in *A. fumigatus* lay on overexpression and/or point mutations in *cyp51A* gene which encodes a 14-α sterol demethylase involved in the ergosterol biosynthesis pathway (Lepesheva and Waterman, 2007; Hargrove et al., 2015).

In this context, two routes of azole resistance acquisition have been described. The acquisition of resistance in the clinical setting – the so called medical route – as a consequence of the in-host drug adaptation of fungus after prolonged azole exposure (Burgel et al., 2012) involves point mutations in the *cyp51A* gene, such as G54 (Diaz-Guerra et al., 2003; Mann et al., 2003), G138 (Howard et al., 2006), M220 (Mellado et al., 2004; Chen et al., 2005), and G448 (Bellete et al., 2010; Krishnan Natesan et al., 2012; Pelaez et al., 2012). Resistance can also been found in isolates from azole-naive patients (Astvad et al., 2014; Pelaez et al., 2015), which suggests an acquisition of azole resistant strains from the environment (Verweij et al., 2007; Snelders et al., 2008). These isolates harbor specific point mutations in *cyp51A* gene together with various size tandem repeat (TR) integrations in the promoter of the gene, which lead to multiazole resistance, and is the most common mechanism of resistance found in environment isolates. The strains most frequently found have a TR integration of 34-bp in the promoter combined with L98H substitution in *cyp51A* gene (Mellado et al., 2007). Strains with 46-bp promoter integration together with Y121F and T289A point mutations (Vermeulen et al., 2012; van der Linden et al., 2013; Pelaez et al., 2015), or only with 53-bp TR integration and without other *cyp51A* substitutions (Hodiamont et al., 2009; Le Pape et al., 2016), have been isolated from the environmental setting as well as from azole-naive patients. The environmental route or the medical route of azole resistance acquisition illustrates the adaptation of isolates to drug selective pressure. However, the selected azole resistance mechanisms are very different as well as are their azole susceptibility profiles. Typing of the isolates could give insight into the dynamics of azole resistance development within an *A. fumigatus* population that includes azole susceptible and resistant isolates with both types of azole resistance mechanisms.

Currently, the short tandem repeat of *A. fumigatus* assay (STR*Af*) based on microsatellite analysis is widely accepted as the reference typing method for this species (de Valk et al., 2005). It consists of a panel of nine STR markers amplified by using multicolor multiplex PCR approaches. However, lack of standardization in the fragment electrophoretic mobility may complicate result comparisons among laboratories. This limitation and the technology required have prompted the development of alternative typing techniques (Balajee et al., 2008). Recently, a novel genotyping method based on hypervariable TRs within exons of surface protein coding genes (TRESP) of three markers has been developed (Garcia-Rubio et al., 2016). TRESP has a considerable discriminatory power (*D* = 0.994), and does not require trained personnel, specific equipment, or software for analysis. In this study we have improved TRESP methodology with the inclusion of a fourth marker to increase its discriminatory power.

The aim of this work was to genotype a large *A. fumigatus* collection of azole susceptible and resistant strains with different resistance mechanisms using both typing methodologies in order to analyze the potential clustering of isolates according to their azole resistance mechanism. In addition, both methodologies were combined to study the improvements in terms of discriminatory power and population structure.

## Materials and Methods

### *Aspergillus fumigatus* Strains

A total of 212 *A. fumigatus* clinical strains isolated from 1997 to 2017 were included in this study (**Table 1**). Among those, 142 were considered unrelated strains since they were isolated from individual patients and were azole susceptible strains. Most of them were from Spain, except for six from Italy, three from United Kingdom, 2 from Netherlands, and one from France. The remaining 70 strains were azole resistant *A. fumigatus* isolates with different azole resistance mechanisms and geographic origins.

**Table 1:** Azole susceptible and resistant *Aspergillus fumigatus* strains included in this study.

*cyp51A* Modifications	Azole susceptibility	No. of strains
Wild type	Susceptible	124
Wild type	Resistant	3
F46Y/M172V/E427K	Susceptible^∗^	13
F46Y/M172V/N248T/D255E/E427K	Susceptible^∗^	4
G54	Resistant	14
M220	Resistant	8
M220/V101F	Resistant	1
N248K	Susceptible	1
G448S	Resistant	1
TR_34_/L98H	Resistant	30
TR_46_/Y121F/T289A	Resistant	12
TR_53_	Resistant	1


*^∗^On the basis of the azole susceptibility breakpoints, these strains cannot be considered azole resistant by CLSI nor EUCAST criteria, but they have higher azole MICs than *cyp51A*-wild type *A. fumigatus* strains (Garcia-Rubio et al., 2018).*

Fungal DNA was extracted from the isolates as described previously (Mellado et al., 2001). Isolates were identified to the species level by PCR amplification and sequencing of ITS region and *β-tubulin* gene (Alcazar-Fuoli et al., 2008). The full coding sequences of *cyp51A*, including its promoter, were amplified and sequenced using the PCR conditions described before (Diaz-Guerra et al., 2003).

### TRESPERG Assay

We used TRESP typing markers and PCR conditions already described (Garcia-Rubio et al., 2016): (i) Afu2g05150 encoding an MP-2 antigenic galactomannan protein (MP2), (ii) Afu6g14090 encoding hypothetical protein with a CFEM domain (CFEM), and (iii) Afu3g08990 encoding a cell surface protein A (CSP). The latter has been the only one extensively used for typing purposes (Balajee et al., 2007). A fourth target, *erg4B* gene (Afu1g07140), which encodes a putative C-24 sterol reductase – named hereafter ERG marker – was added to improve discriminatory power. ERG repeat sequences are formed mainly by 12-mer repeats, coding a particular sequence of amino acids (Supplementary Table S1). The proposed typing nomenclature for the ERG marker followed the one described for CSP’s structure (Balajee et al., 2007), in line with the other TRESP markers (Garcia-Rubio et al., 2016). The typing method concerning the four markers will from now be called TRESPERG assay.

Primers used to partially amplify *erg4B* gene were erg4B_P1 (5′-ATGACTGTCACACGCTCC-3′) and erg4B_P2 (5′-TAGACGGCACCAATCCAC-3′). Amplicon size was variable, between 608 and 754 bp, depending on the number of repeats in each strain. The PCR amplification was performed using the same equipment as in TRESP typing (Garcia-Rubio et al., 2016) as follows: 1 cycle of 5 min at 94°C and then 35 cycles of 30 s at 94°C, 45 s at 58°C, and 2 min at 72°C; and 1 final cycle of 5 min at 72°C. The PCR products were purified using Illustra Exoprostar 1-step (GE Healthcare Life Science, United Kingdom) and both strands were sequenced using the same PCR amplification primers.

Sequences were assembled using the Lasergene software package (DNAStar, Inc., United States) and aligned using MAFFT version 7 (Katoh et al., 2002). The final genotype was obtained after combining the four alleles of each target. In TRESPERG typing, genotypes were considered identical only when they showed the same alleles for all four loci.

### STR*Af* Assay

PCR for amplification of the nine STR*Af* loci in three multiplexed reactions were performed as previously described (de Valk et al., 2005). Electropherograms were analyzed using GeneMapper (version 4.0) software (Thermo Fisher Scientific, Spain). Genotypes were considered identical when they showed the same alleles for all nine loci (Balajee et al., 2008; Guinea et al., 2011).

### Discriminatory Power and Genotypic Diversity Analysis

The discriminatory power was calculated with the Simpson’s index of diversity (*D*) using unrelated strains as previously described (Hunter and Gaston, 1988) therefore only unrelated azole susceptible strains were used.

For both typing assays, genotypic diversity and clustering analysis were determined by the Unweighted Pair Group Method Using Arithmetic Averages (UPGMA) and minimum spanning trees (MST) were performed using BioNumerics (version 6.0.1) software (Applied Maths, Belgium).

## Results

### TRESP Improvement: New TRESPERG Typing Assay

The nucleotide and amino acid sequence of each repeat type are described in Supplementary Table S1 and the different ERG alleles identified among the global *A. fumigatus* population and the TR succession forming each allele are shown in Supplementary Table S2.

New MP2 and CFEM genotypes were found when all strains were characterized. Three new MP2 variants, designated here as m1.10, m6.4, and m6.5 were identified (Supplementary Table S3). In the m6.5 MP2 allele, a new repeat sequence was detected (GAGACCTCCACTCCTACCGAGACCACTACCACTCCTACC, named r27). Also, two new CFEM types, c22 and c23, were described among these isolates and were added to the primary set (Supplementary Table S4).

**FIGURE 1:**
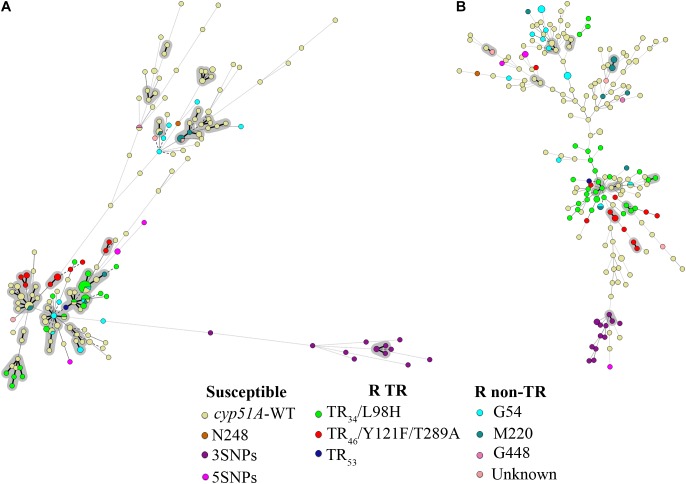
Minimum spanning tree (MST) showing the genotypic diversity of azole susceptible and resistant *Aspergillus fumigatus* isolates. **(A)** TRESPERG MST and **(B)** STR*Af* MST. *Each circle* shows a unique genotype, and its size the number of strains belonging to the same genotype. *Connecting lines* between *circles* show the similarity between genotypes: *solid* and *bold* (shaded in gray) indicate only one marker difference, a *solid line* indicates differences in two markers, and *dashed lines* for differences in three or more markers. The *different colors* of the *circles* indicate the *cyp51A* modifications, grouped in azole susceptible, azole resistant with TRs (R TRs), and azole resistant with punctual *cyp51A* mutations (R non-TR).

TRESPERG revealed a total of 119 different genotypes among the 142 azole susceptible unrelated *A. fumigatus* strains. In summary 20 CSP, 30 MP2, 24 CFEM, and 17 ERG genotypes were identified. Using these results, the discriminatory power (*D*) of the technique was 0.9972 (**Table 2**). When the 212 strains were considered, the number of TRESPERG genotypes increased to 156, and the number of genotypes for each marker also increased: 22 CSP, 32 MP2, 25 CFEM, and 18 ERG. In this case, the *D* decreased to 0.9933 (**Table 2**). The MST (**Figure 1A**) showed high genotypic variability among the *A. fumigatus* isolates, where seven genetic clusters were identified, consistent with the TRESPERG dendrogram (Supplementary Figure S1).

**Table 2:** Number of different genotypes and Simpson’s index of diversity (*D*) for both typing methodologies (TRESPERG and STR*Af*), a STR*Af* variant (STR*Af* without M3), and the combination of both methods (Combined).

	Susceptible unrelated strains		Resistant strains				Overall	
	(*n* = 142)		(*n* = 70)				(*n* = 212)	
			TR with *cyp51A* point mutations		*cyp51A* point mutations			
			(*n* = 43)		(*n* = 27)			
		
Markers	No. GT^∗^	*D*	No. GT^∗^	*D*	No. GT^∗^	*D*	No. GT^∗^	*D*
TRESPERG	119	0.9972	27	0.9491	20	0.9715	156	0.9933
STR*Af*	135	0.9993	41	0.9978	20	0.9744	195	0.9992
STR*Af* without M3	128	0.9985	34	0.9856	19	0.9630	177	0.9979
Combined	136	0.9994	42	0.9989	24	0.9915	201	0.9995


*No. GT^∗^, number of genotypes.*

### STR*Af* Analysis

The same 142 unrelated strains were used for STR*Af* typing analysis, obtaining 135 different genotypes. When all the strains were included, the number of genotypes increased to 195. The *D* value of the STR*Af* assay, calculated using only unrelated strains, was 0.9993, while it was 0.9992 when all isolates were included in the analysis (**Table 2**). STR*Af* showed higher genotypic variability than TRESPERG (**Figure 1B**), but the *A. fumigatus* population could be distributed in six clusters instead of seven (Supplementary Figure S2).

### Genotypic Diversity: Typing Azole Resistance

The set of 70 *A. fumigatus* azole resistant strains included: (i) strains only with *cyp51A* single point mutations (G54, M220, or G448) and (ii) strains with tandem integrations and/or *cyp51A* mutations (TR_34_/L98H, TR_46_/Y121F/T289A, or TR_53_). There were three azole resistant wild type *cyp51A* strains that were included with isolates from the *cyp51A* single point mutations group for the analysis. When both typing methods were considered individually, there were some differences in terms of how the *A. fumigatus* population was clustered. In TRESPERG assay, seven clusters were identified as said before (**Figure 1A** and Supplementary Figure S1). The azole resistant isolates which harbored a TR resistant mechanism grouped together in only one cluster, while azole susceptible isolates were distributed in five clusters (**Figure 1** and Supplementary Figure S1). In the dendrogram, there were two different clusters with only *cyp51A*-WT strains, two more independent clusters: one with *cyp51A*-5SNPs (F46Y, M172V, N248T, D255E, and E427K) and another with *cyp51A*-3SNPs (F46Y, M172V, and E427K) strains (Garcia-Rubio et al., 2018), two more clusters with *cyp51A*-WT strains and single point mutation strains, and finally a greater cluster where TR are clustered together along with some *cyp51A*-WT and point mutation resistant strains.

However, when the STR*Af* assay was used, the resistant strains – independently of their resistance mechanisms – were widely spread as susceptible isolates (**Figure 1B** and Supplementary Figure S2). In the dendrogram, a total of six genetic clusters were found. TR azole resistant strains were distributed in three different clusters and azole resistant strains with *cyp51A* point mutations were spread in four clusters. With TRESPERG, *cyp51A*-3SNPs (F46Y, M172V, and E427K) strains were grouped all in one cluster, whereas *cyp51A*-5SNPs (F46Y, M172V, N248T, D255E, and E427K) strains were distributed in two different and distant clusters.

### Combined TRESPERG STR*Af* Analysis

**FIGURE 2:**
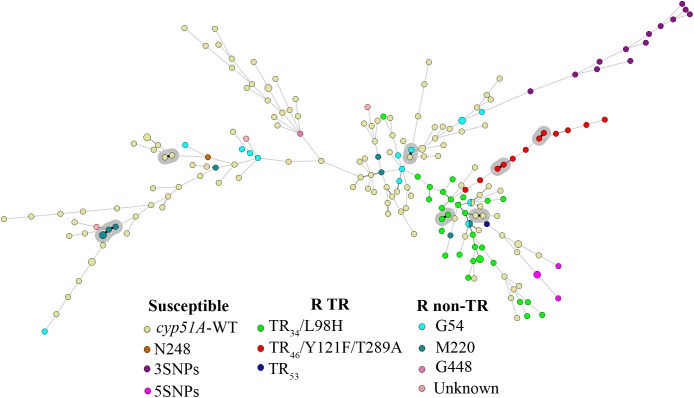
Minimum spanning tree showing the genotypic diversity of azole susceptible and resistant *Aspergillus fumigatus* isolates combining TRESPERG and STR*Af* markers. *Each circle* shows a unique genotype, and its size the number of strains belonging to the same genotype. *Connecting lines* between *circles* show the similarity between genotypes: *solid* and *bold* (shaded in gray) indicate only one marker difference, a *solid line* indicates differences in two markers, and *dashed lines* for differences in three or more markers. The *different colors* of the *circles* indicate the *cyp51A* modifications, grouped in azole susceptible, azole R TR, and azole resistant with punctual *cyp51A* mutations (R non-TR).

In order to determine how much the *D* would be improved combining both genotyping techniques, the nine STR*Af* markers were combined with the four TRESPERG ones. The *D* obtained using only unrelated strains was 0.9994, while it was 0.9995 when all isolates were included in the analysis (**Table 2**). The combination of both typing assays analysis was represented in a MST (**Figure 2**) and in a dendrogram (Supplementary Figure S3), showing all TR strains were grouped in only one cluster as in individual TRESPERG analysis.

## Discussion

Many different molecular methods have been described for *A. fumigatus* typing purposes, such as random amplified polymorphic DNA (RAPD) (Loudon et al., 1993), amplified fragment length polymorphism analysis (AFLP) (Warris et al., 2003), restriction fragment length polymorphism analysis (RFLP) (Neuveglise et al., 1996), microsatellite length polymorphism (MLP) (Bart-Delabesse et al., 1998), retrotransposon insertion-site context (RISC) typing (de Ruiter et al., 2007), and multilocus sequence typing (MLST) (Bain et al., 2007). However, the discriminatory power values are variable among these techniques and some of them have a poor inter-laboratory reproducibility. The current gold standard method is the STR*Af* assay, based on microsatellite genotyping, which is able to discriminate an extensive amount of *A. fumigatus* unrelated isolates with high discriminatory power (de Valk et al., 2005). STR*Af* assay is a potent typing system and a suitable molecular tool to support outbreak and epidemiological investigations (Balajee et al., 2008). Microsatellite data, if properly gathered, are straightforward to analyze. However, it involves some methodological disadvantages (de Valk et al., 2007; Klaassen and Osherov, 2007; Klaassen, 2009a), which has encouraged the development of novel typing techniques using more accessible approaches while keeping a good discriminatory power. In this context, this work aimed to test two different typing methods with the same set of *A. fumigatus* strains, in order to obtain perfectly comparable discriminatory power values. As an interesting typing alternative, TRESPERG methodology has a good reproducibility with a high discriminatory power (*D* = 0.9972), although less than the STR*Af* assay (*D* = 0.9993) against the same set of unrelated *A. fumigatus* strains. In published guidelines for genetic typing methodologies, some authors established 0.90 as a suitable discriminatory power (Hunter and Gaston, 1988), while others recommended that this value was greater than or equal to 0.95 (van Belkum et al., 2007). Both methods, STR*Af* and TRESPERG, beat these discriminatory power values. In previous works, other authors have determined that *D* for STR*Af* assay was 0.999 (de Valk et al., 2005), 0.984 (Guinea et al., 2011) or 0.969 (Shen et al., 2018), depending on the set of strains. TRESP typing has been less used and the discriminatory values ranged from 0.994 (Garcia-Rubio et al., 2016) to 0.939 (Shen et al., 2018). In all these works, the number of strains and the set of isolates varied between studies. Since the discriminatory power must be calculated using unrelated strains, the specific set of *A. fumigatus* population strains used to determine the *D* of each technique is essential. In this work, the number of genotypes among the *A. fumigatus* unrelated population obtained by STR*Af* was higher than the genotypes obtained by TRESPERG (135 vs. 119, respectively). When the 31 genotypes that were common for more than one isolate in TRESPERG typing were considered, STR*Af* was able to discriminate nineteen of them in different genotypes. On the contrary, among the 15 genotypes with more than one isolate in STR*Af* assay, TRESPERG was able to discriminate only three of them. In this context, STR*Af* has a superior *D* value and is able to discriminate more genotypes than TRESPERG.

*A. fumigatus* has been the subject of many studies showing that this species is genetically very diverse and have a panmictic population structure using both microsatellite markers or whole genome sequence data (Abdolrasouli et al., 2015; Ashu et al., 2017). Both typing methodologies (TRESPERG and STRAf) were used to compare the genetic diversity and the clustering of azole resistant *A. fumigatus* strains isolated from different geographical locations (Spain, United Kingdom, Netherlands, Denmark, and France). Most of the clusters contained isolates from different origins with both typing methods (Supplementary Figures S1, S2). Similar results have been described in a recent broad study which typed a huge number of isolates from 13 countries in four continents using STR*Af* (Ashu et al., 2017). In this study, the *A. fumigatus* population grouped into eight genetic clusters, with seven of the eight clusters showing wide geographic distributions. However, there were differences regarding the clustering of the azole resistant population between the two methodologies tested in our work. Generally, there is strong evidence that non-TR strains (azole susceptible and *cyp51A* single point mutation resistance strains) have a greater genetic diversity than TR resistant isolates, since the expansion of these TR resistant strains at a local level is predominantly clonal (Chowdhary et al., 2012; Abdolrasouli et al., 2015; Chang et al., 2016; Ashu et al., 2017; Garcia-Rubio et al., 2017; Wang et al., 2017). Specifically, *cyp51A* point mutations are more commonly isolated from patients undergoing long periods of azole treatment (Burgel et al., 2012), so it can be expected that they did not have much more in common unless they came from sequential isolates of the same patient (Dannaoui et al., 2001; Mortensen et al., 2011). It is important to highlight that G54E *cyp51A* point mutation has also been described in both clinical and environmental isolates, and were distributed in different genotypic clusters when the STR*Af* assay was used to genotype the isolates (Sharma et al., 2015). In addition, these isolates were cross-resistant to agricultural fungicides, which suggests that this resistance mechanism can also be acquired from the environment and coexist with TR isolates (Sharma et al., 2015).

Our study supported the idea that non-TR strains have a greater genetic diversity than TR azole resistant isolates by the fact that *cyp51A* point mutated resistant and susceptible strains were distributed across most of the clusters using both typing approaches. Moreover, all TR azole resistant strains (TR_34_/L98H, TR_46_/Y121F/T289A, and TR_53_) were located in one specific cluster in TRESPERG typing, which reinforces the previously suggested idea that TR resistance mechanisms have developed from a reduced set of clonally related strains with shorter genetic distances among themselves (Snelders et al., 2009; Camps et al., 2012). Another work has obtained similar results to those obtained using TRESPERG, with TR isolates located all together in one specific cluster while non-TR were distributed across all the clusters formed (Klaassen et al., 2012). However, this result was not obtained using STR*Af* assay, where TR azole resistant strains were distributed in three different clusters (Supplementary Figure S2). Similarly, some authors have described this dispersed structure in the TR azole resistant *A. fumigatus* population using STR*Af* as genotyping method (Ashu et al., 2017; Chen et al., 2018), although in some studies this may be due to the lack of differentiation between azole resistance mechanisms (Ashu et al., 2017).

The way in which each typing method clustered the isolates of this *A. fumigatus* population depends on the genetic markers used and how they express the genetic relationships among them. In this context, the results seem to indicate that in this set of strains, TRESPERG markers clustered TR azole resistant strains in a better manner than STR*Af* assay did. Indeed, many authors have described these strains as genetically more closely related, as indicated by TRESPERG (Snelders et al., 2009; Camps et al., 2012; Chowdhary et al., 2012; Abdolrasouli et al., 2015; Chang et al., 2016; Ashu et al., 2017; Wang et al., 2017). A possible explanation for the disagreement between TRESPERG and STR*Af* could be that microsatellites are extremely high discriminatory as a consequence of its inherent instability. Therefore, the interpretation of genotypically different isolates should be analyzed carefully and this instability should be taken into account (Klaassen, 2009b). Since STR*Af* M3 has been described as the most variable and discriminatory marker (de Valk et al., 2005), the STR*Af* assay was also analyzed excluding this marker. The *D* of the technique continued to be very high (**Table 2**), which means STR*Af* could be used for typing purposes even if M3 marker is removed from the assay. Actually, the STR*Af* MST without M3 (Supplementary Figure S4) was more similar to the MST obtained by TRESPERG (**Figure 1A**), with all azole resistant TR strains located in only one cluster.

Moreover, the combined analysis of STR*Af* and TRESPERG assays (**Table 2**) resulted in eight clusters (Supplementary Figure S3) and all TR strains grouped together (**Figure 2**). Again, this supports the hypothesis that they could have developed from a reduced set of clonally related strains and therefore could have shorter genetic distances compared to other *A. fumigatus* strains. Interestingly, all TR_46_/Y121F/T289A emerged from only one branch of the MST (**Figure 2**).

It is worth pointing out that the TR resistant strains used in this analysis came from different countries in Europe and were isolated over a long period of time. Their genetic distribution could be consistent with the three possibilities suggested by other authors: (i) the localized and differential azole drug exposure between regions leads to the distinctive clonal expansion of triazole resistant genotypes; (ii) the special predisposition/likelihood of this cluster to develop triazole resistance; (iii) TR strains could be more receptive than those in other clusters in accepting triazole-resistant genes via mating and recombination (Ashu et al., 2017). Future studies using methodologies with higher resolution, such as whole-genome sequencing, will help to unravel the *A. fumigatus* population structure, giving insight into the dynamics of resistance

In summary, both TRESPERG and STR*Af* assays have sufficient discriminatory power and both are perfectly reliable for epidemiological investigations showing comparable typing results from the *A. fumigatus* population under analysis. STR*Af* offered a higher discriminatory power compared to TRESPERG. However, the latter is able to better group azole resistant strains depending on their resistance mechanisms. Also, its methodological simplicity allows for easy integration into any clinical microbiology laboratory, fulfilling all the needs of a suitable typing assay.

## Accession Numbers

Sequences from each new genotype described in the TRESPERG analysis have been submitted to GenBank under accession numbers MH607379 to MH607402.

## Author Contributions

EM and RG-R conceived and designed the experiments. RG-R and AG performed the experiments. RG-R, PE, JG, and EM analyzed the data. JG and EM contributed materials. All authors wrote the paper, discussed the results, and agreed to be accountable for all aspects of the work in ensuring accuracy.

## Conflict of Interest Statement

The authors declare that the research was conducted in the absence of any commercial or financial relationships that could be construed as a potential conflict of interest.
