# Supplementary figures and images for: Comparison of Two Highly Discriminatory Typing Methods to Analyze Aspergillus fumigatus Azole Resistance
Source: Front Microbiol. 2018 Jul 20;9:1626. doi: 10.3389/fmicb.2018.01626 (PMC6062602; doi:10.3389/fmicb.2018.01626)

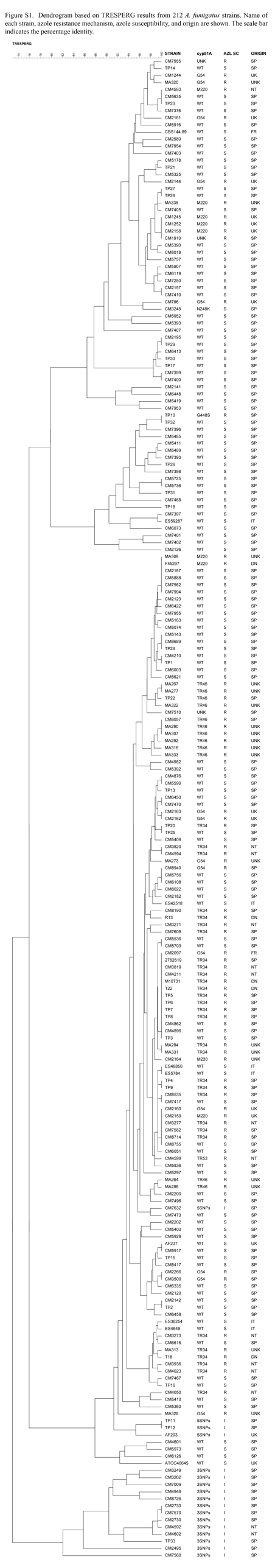

Supplement: Supplementary file 1 [file Image_1.TIF]

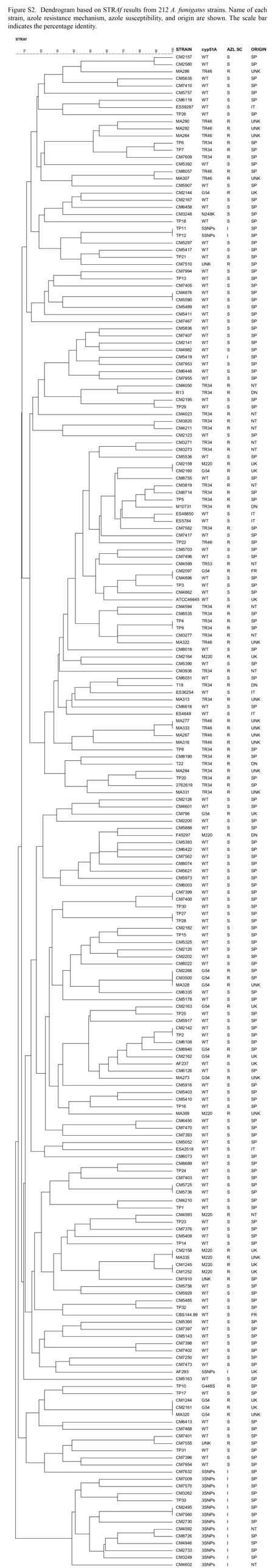

Supplement: Supplementary file 2 [file Image_2.TIF]

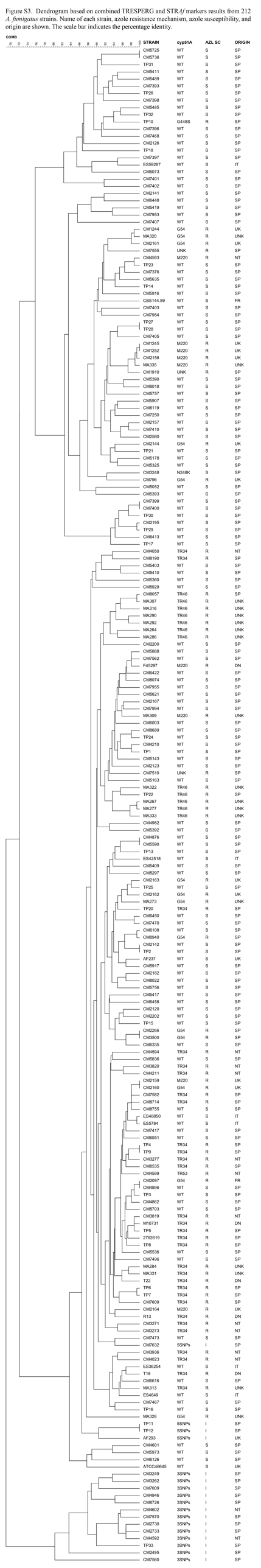

Supplement: Supplementary file 3 [file Image_3.TIF]

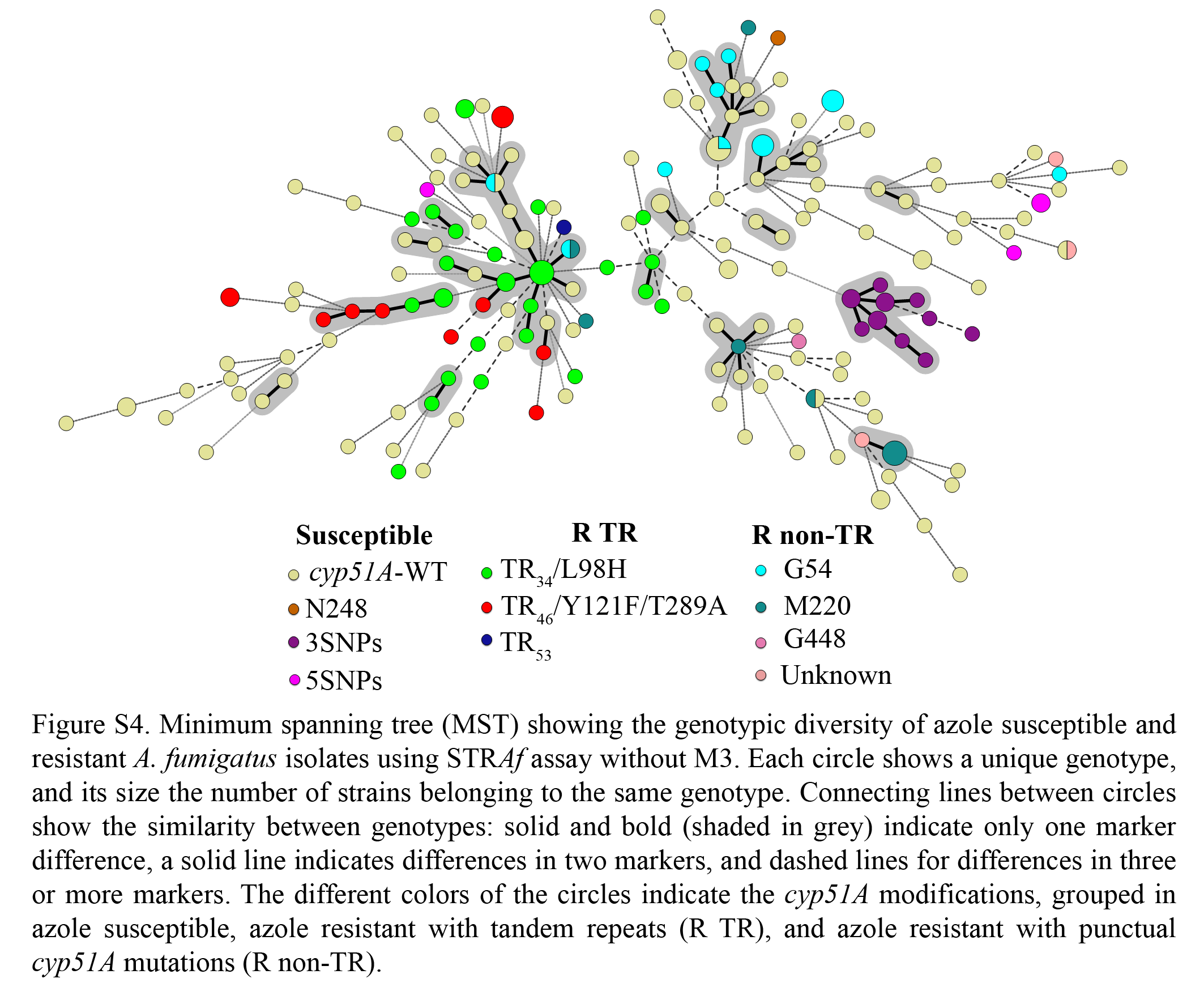

Supplement: Supplementary file 4 [file Image_4.TIF]
