# Supplementary material for: Comparison of Two Highly Discriminatory Typing Methods to Analyze Aspergillus fumigatus Azole Resistance
Source: Front Microbiol. 2018 Jul 20;9:1626. doi: 10.3389/fmicb.2018.01626 (PMC6062602; doi:10.3389/fmicb.2018.01626)
Supplement: Supplementary file 5 [file Table_1.DOCX]

Table S1. ERG repeat types: nucleotide and amino acid sequences identified among 212 *A. fumigatus* isolates.

| **ERG Repeat type** | **Amino acid sequence** | **Repeat sequence** |
| --- | --- | --- |
| r01 | HING | cacatcaatgga |
| r02 | NTNG | aacaccaatgga |
| r03 | HTNG | cacaccaatgga |
| r04 | HTNG | cataccaatgga |
| r05 | HTNG | cacaccaatggt |
