# Supplementary material for: Comparison of Two Highly Discriminatory Typing Methods to Analyze Aspergillus fumigatus Azole Resistance
Source: Front Microbiol. 2018 Jul 20;9:1626. doi: 10.3389/fmicb.2018.01626 (PMC6062602; doi:10.3389/fmicb.2018.01626)
Supplement: Supplementary file 6 [file Table_2.DOCX]

Table S2. ERG alleles identified among 212 *A. fumigatus* isolates.

| **ERG Allele** | **Tandem repeat succession** |
| --- | --- |
| e01 | 01-02-05 |
| e02 | 01-02-02-03-05 |
| e03 | 01-02-02-03-05* |
| e04 | 03-03-05 |
| e05 | 01-02-03-03-05 |
| e06 | 01-02-02-03-03-05 |
| e07 | 01-02-02-03-03-03-05 |
| e08 | 01-02-03-03-03-03-05 |
| e09 | 01-02-02-03-03-03-03-05 |
| e10 | 01-02-02-04-03-03-03-05* |
| e11 | 01-02-02-03-03-03-03-03-05 |
| e12 | 01-02-02-04-03-03-03-03-05* |
| e13 | 01-02-02-03-03-03-03-03-03-05 |
| e14 | 01-02-02-04-03-03-03-03-03-05* |
| e15 | 01-02-02-03-03-03-03-03-03-03-05 |
| e16 | 01-02-02-03-03-03-03-03-03-03-03-05 |
| e17 | 01-02-02-03-03-03-03-03-03-03-03-03-05 |
| e18 | 01-02-02-03-03-03-03-03-03-03-03-03-03-05 |
| e19 | 01-02-02-03-03-03-03-03-03-03-03-03-03-03-05 |

***** Synonymous mutations g27a, G9; c205t; a351c, T117.
