# Supplementary material for: Comparison of Two Highly Discriminatory Typing Methods to Analyze Aspergillus fumigatus Azole Resistance
Source: Front Microbiol. 2018 Jul 20;9:1626. doi: 10.3389/fmicb.2018.01626 (PMC6062602; doi:10.3389/fmicb.2018.01626)
Supplement: Supplementary file 7 [file Table_3.DOCX]

Table S3. MP2 alleles identified among 212 *A. fumigatus* isolates.

| **MP2 Alleles** | **Tandem repeat succession** |
| --- | --- |
| m1.1 | 01-02-[P-P-P]-01-03-04-05-06-------------------------------------07-08-[P-P-P]-09-------------04-10-11-[P-P] |
| m1.2 | 01-02-[P-P-P]-01-----04-05-06-------------------------------------07-08-[P-P-P]-09-------------04-10-11-[P-P] |
| m1.3 | 01-02-[P-P-P]-01---------05-06-------------------------------------07-08-[P-P-P]-09-------------04-10-11-[P-P] |
| m1.4 | 01-02-[P-P-P]-------------05-06-------------------------------------07-08-[P-P-P]-09-------------04-10-11-[P-P] |
| m1.5 | 01-02-[P-P-P]-01-----04-----06-------------------------------------07-08-[P-P-P]-09-------------04-10-11-[P-P] |
| m1.6 | 01-02-[P-P-P]-01---------05-06-------------------------------------07-08-[P-P-P]-09-----------------10-11-[P-P] |
| m1.7 | ----02-[P-P-P]-01-03-04-05-06-------------------------------------07-08-[P-P-P]-09-------------04-10-11-[P-P] |
| m1.8 | ------------------01-03-04-05-06-------------------------------------07-08-[P-P-P]-09-------------04-10-11-[P-P] |
| m1.9 | 01-02-[P-P-P]-01-03-04-05-06-------------------------------------07-08-[P-P-P]---------01-03-04-10-11-[P-P] |
| m1.10* | ------------------01-03-----05-06-------------------------------------07-08-[P-P-P]-09-------------04-10-11-[P-P] |
| m2.1 | 01-02-[P-P-P]-01-03-04-----06-12-13-4-6-12-02-[P-P-P]-14--07-08-[P-P-P]-09-------------04-10-11-[P-P] |
| m2.2 | 01-02-[P-P-P]-01-03-04-----06-12--------------02-[P-P-P]-14--07-08-[P-P-P]-09-------------04-10-11-[P-P] |
| m3.1 | 03------------------------------------------------------------------------------------------------------03-22-[P-P] |
| m3.2 | 03--------------------------------------------------15-20-15-S-21-----15--------------------------03-22-[P-P] |
| m3.3 | 03--------------------------------05-03-----------15-20-15-S-21-----15--------------------------03-22-[P-P] |
| m3.4 | 03-15----------------------------05-03-----------15-20-15-S-21-----15--------------------------03-22-[P-P] |
| m3.5 | 03-15-20-15-S-21-15-03-15-05-03-----------15-20-15-S-21-----15--------------------------03-22-[P-P] |
| m3.6 | 03-15----------------------------05-03-----------15-20-15-S-21-06-15-20-15-S-21-15-S-21-03-22-[P-P] |
| m3.7 | 03-15-20--------------------------------------26-15-20-15-S-21-----15--------------------------03-22-[P-P] |
| m4.1 | ---------------------------03----05-03-----------15-20-15-S-21------15-20-15-S-21-15-------03-17-[P-P] |
| m4.2 | ---------------------------03----05-03-----------15-20-15-S-21------15--------------------------03-17-[P-P] |
| m5.1 | 03-15-05-03-15------------------------------------S-S-S-16-15-03--15------------------------------17-[P-P] |
| m5.2 | 03-15-05-03-15-------------------------------------- S-S-16-15-03--15------------------------------17-[P-P] |
| m5.3 | 03-15-05-03-15-----------------------------------------S-16-15-03--15-------------------------------17-[P-P] |
| m5.4 | 03-15-05-03-15--------------------------------------------16-15-03--15-------------------------------17-[P-P] |
| m5.5 | 03-15-05-03-15--------------------------------------------16-15-16--15-03--15----------------------17-[P-P] |
| m5.6 | 03-15-05-03-15-------------------------------------------------------------------------------------------17-[P-P] |
| m5.7 | 03----------------------------------------------25---------------15-03--15-------------------------------17-[P-P] |
| m6.1 | 03-15-05-03-15-05-03-15-05-03-15-05-03-15-05----16-15----------------------------------------17-[P-P] |
| m6.2 | 03-15-05-03-15-05-03-15-05-03-15--------------------16-24-16-15--------------------------------17-[P-P] |
| m6.3 | 03-15--------------------------------------------------------16-15-16-15--------------------------------17-[P-P] |
| m6.4* | 03-15-------------------------------------------------------------------------------------------------------17-[P-P] |
| m6.5* | 03-15-05-03-15-05-03-15-05-27-15-05-03-15-05----16-15----------------------------------------17-[P-P] |
| m7.1 | 18--------08-[P-P-P]-19---------------02-[P-P-P]-01-03-07-08-[P-P-P]-07--------------------23-11-[P-P] |
| m8.1 | ---------------------------------------03-02-[P-P-P]-01-03-07-08-[P-P-T]-07-02-[P-P-P]-19-23-11-[P-P] |
| m9.1 | -------------------------------------------------------------03-07-08-[P-P-T]-07-------------------23-11-[P-P] |
| m10.1 | 07--------08-[P-P-P]---------------------------------01-03-07-08-[P-P-T]-07-------------------23-11-[P-P] |

*New MP2 genotypes described in this work. P, proline.
