# Supplementary material for: Comparison of Two Highly Discriminatory Typing Methods to Analyze Aspergillus fumigatus Azole Resistance
Source: Front Microbiol. 2018 Jul 20;9:1626. doi: 10.3389/fmicb.2018.01626 (PMC6062602; doi:10.3389/fmicb.2018.01626)
Supplement: Supplementary file 8 [file Table_4.DOCX]

Table S4. CFEM alleles identified among 212 *A. fumigatus* strains.

| **CFEM Alelles** | **Tandem repeat succession** | **Nº R11** | **Nº TRs (bp)** | **Nº R12** |
| --- | --- | --- | --- | --- |
| c01 | 06-01-01-01-02-02-01-03-03-04-03-05-----------------04-03-03-03-06-01-04-03-05-07-03-04-03-08-01-03-09-10 | 6 | 21 | 4 |
| c02 | 06-01-01-01-----02-01-03-03-04-03-05-----------------04-03-03-03-06-01-04-03-05-07-03-04-03-08-01-03-09-10 | 7 | 21 | 4 |
| c03 | 06-01-01-01-----02-01-03-03-04-03-05-----------------04-03-03-03-06-01-04-03-05-07-03-04-03-08-01-03-09-10 | 5 | 21 | 4 |
| c04 | 06-01-01-01-----02-01-03-03-04-03-05-----------------04-03-03-03-06-01-04-03-05-07-03-04-03-08-01-03-09-10 | 4 | 21 | 4 |
| c05A | 06-01-01-01-----02-01-03-03-04-03-05-----------------04-03-03-03-06-01-04-03-05-07-03-04-03-08-01-03-09-10 | 3 | 21 | 5 |
| c05B^#^ | 06-01-01-01-----02-01-03-03-04-03-05-----------------04-03-03-03-06-01-04-03-05-07-03-04-03-08-01-03-09-10 | 3 | 21 | 5 |
| c06 | 06-01-01-01-----02-01-03-03-04-03-05-----------------04-03-03-03-06-01-04-03---------------------08-01-03-09-10 | 3 | 21 | 5 |
| c07 | 06-01-01-01-----02-01-------------03-05-----------------04-03-03-03-06-01-04-03-05-07-03-04-03-08-01-03-09-10 | 3 | 21 | 4 |
| c08A | 06-01-01-01-----02-01-03-03-04-03-05-----------------04-03-03-03-06-01-04-03-05-07-03-04-03-08-01-03-09-10 | 3 | 21 | 4 |
| c08B^$^ | 06-01-01-01-----02-01-03-03-04-03-05-----------------04-03-03-03-06-01-04-03-05-07-03-04-03-08-01-03-09-10 | 3 | 21 | 4 |
| c09 | 06-01-01-01-----02-01-03-03-04-03-05-----------------04-03-03-03-06-01-04-03-05-07-03-04-03-08-01-03-09-10 | 2 | 21 | 4 |
| c10^&^ | 06-01-01-01-----02-01-03-03-04-03-05-----------------04-03-03-03-06---------------------------------------------09-10 | 2 | 21 | 4 |
| c11^%^ | 06-01-01-01-----02-01-03-03-04-03-05-------------------------03-06-----------------------------------------01-03-09-10 | 4 | 21 | 4 |
| c12 | 06-01-01-01-----02-01-----------------------------------------03-03-03-06-01-04-03-05-07-03-04-03-08-01-03-09-10 | 3 | 21 | 4 |
| c13^%^ | 06-01-01-01-----02-01-03-03-04-03-05-----------------------------------------------------------------------------03-09-10 | 2 | 21 | 4 |
| c14^%^ | 06-01-01-01-----02-01-03-03-04-03-05-----------------------------------------------------------------------------03-09-10 | 3 | 21 | 4 |
| c15 | 06-01-01-01-----02-01-03-03-04-03-05-----------------------------03-06-------------------------------------01-03-09-10 | 6 | 21 | 4 |
| c16 | 06-01-01-01-----02-01-03-03-04-03-05-----------------04-03-03-03-06-13-04-03-05-07-03-04-03-08-01-03-09-10 | 2 | 21 | 4 |
| c17 | 06-01-01-01-----02-01-03-03-04-03-05-----------------04-03-03-03-06-01-04-03-05-07-03-04-03-08-01-03-09-10 | 2 | 21 | 5 |
| c18 | 06-01-01-01-----02-01-03-03-04-03-05-----------------04-03-03-03-06-01-04-04-05-07-03-04-03-08-01-03-09-10 | 2 | 21 | 4 |
| c19 | 06-01-01-01-01-02-01-03-03-04-03-05-04-03-03-03-04-03-03-03-06-------------------------------------01-03-09-10 | 4 | 21 | 3 |
| c20 | 06-01-01-01-----02-01-03-03-04-03-------------------------03-03-06-----01-04-03-05-07-03-04-03-08-01-03-09-10 | 3 | 21 | 4 |
| c21 | 06-01-01-01-----02-01-03-03-04-03-05-------------------------03-06-----------------------------------------01-03-09-10 | 3 | 21 | 4 |
| c22* | 06-01-01-01-----02-01-03-03-04-03-05-----------------04-03-03-03-06-01-04-03---------------------08-01-03-09-10 | 2 | 21 | 5 |
| c23* | 06-01-01-01-----02-01-03-03-04-03-05-----------------04-03-----------------04-03---------------------08-01-03-09-10 | 2 | 21 | 4 |

#SNP122 g/a, %SNP155 a/g, &SNP685 t/a, and $SNP815 a/t. *New CFEM genotypes described in this work. Positions for single nucleotide polymorphisms (SNP) were determined according to the whole genome sequence of *Aspergillus fumigatus* Af293 (GenBank accession number CM000174.1), from 3593051 to 3594095 bp.
